# Correction: A Fatty Acid Based Bayesian Approach for Inferring Diet in Aquatic Consumers

**DOI:** 10.1371/journal.pone.0134258

**Published:** 2015-07-28

**Authors:** The PLOS ONE Staff

## Notice of Republication

This article was republished on July 9, 2015, to replace text and equations in the Methods section that were lost during the production process. Please download this article again to view the correct version. The originally published, uncorrected article and the republished, corrected article are provided here for reference.

## Supporting Information

S1 File Originally published, uncorrected article. (PDF)

S2 File Republished, corrected article. (PDF)

## References

[1] Galloway AWE, Brett MT, Holtgrieve GW, Ward EJ, Ballantyne AP, Burns CW, et al (2015) A Fatty Acid Based Bayesian Approach for Inferring Diet in Aquatic Consumers. PLoS ONE 10(6): e0129723 doi: 10.1371/journal.pone.0129723 26114945 10.1371/journal.pone.0129723 PMC4482665

