# Supplementary material for: A Fatty Acid Based Bayesian Approach for Inferring Diet in Aquatic Consumers
Source: PLoS One. 2015 Jun 26;10(6):e0129723. doi: 10.1371/journal.pone.0129723 (PMC4482665; doi:10.1371/journal.pone.0129723)
Supplement: S1 Fig — (DOC) [file pone.0129723.s001.doc]

**S1 Fig. The distribution of normalized z-scores for the fatty acid data used to generate our resource library.** This plot shows our data for the main FAs are somewhat skewed, but not dramatically so. For example, the median for these data is only 0.15 SDs different from the mean, which corresponds to a 4% bias about the central tendency. Further, the tails in this case are fairly well behaved, for example, with 250 observations we would expect to have 11 cases greater than 2 SDs from the mean and we actually had 7 cases. Issues with non-normality typically arise when datasets are strongly skewed, so we believe our data are robust in this regard.
