# Supplementary material for: A Fatty Acid Based Bayesian Approach for Inferring Diet in Aquatic Consumers
Source: PLoS One. 2015 Jun 26;10(6):e0129723. doi: 10.1371/journal.pone.0129723 (PMC4482665; doi:10.1371/journal.pone.0129723)
Supplement: S1 Table — (DOC) [file pone.0129723.s003.doc]

**S1 Table. The FA composition of phytoplankton monocultures and *Daphnia* fed these monocultures.** These data are reported as the percent of total fatty acids. The values reported in the lower panel are the respective r^2^ values between the two dataset. The values in bold font indicate the fatty acids molecules that best differentiated between the different diet types.
