# Supplementary material for: A Fatty Acid Based Bayesian Approach for Inferring Diet in Aquatic Consumers
Source: PLoS One. 2015 Jun 26;10(6):e0129723. doi: 10.1371/journal.pone.0129723 (PMC4482665; doi:10.1371/journal.pone.0129723)
Supplement: S2 Table — (DOC) [file pone.0129723.s004.doc]

**S2 Table. The outcomes for our test of how two different priors, *i.e.,* the uniform Dirichlet distribution (α =1) and α = dunif(0,100), affected model outputs.** These priors were tested same datsets for SI and FA based analyses using both MixSIR and SIAR. The 2.5 and 97.5 results represent the end-points for the 95% credible intervals.
