# Supplementary material for: Correction: A Fatty Acid Based Bayesian Approach for Inferring Diet in Aquatic Consumers
Source: PLoS One. 2015 Jul 28;10(7):e0134258. doi: 10.1371/journal.pone.0134258 (PMC4517787; doi:10.1371/journal.pone.0134258)
Supplement: S2 File — (PDF) [file pone.0134258.s002.pdf]

PLOS ONE

RESEARCH ARTICLE

A Fatty Acid Based Bayesian Approach for Inferring Diet in Aquatic Consumers

Aaron W. E. Galloway[1☯]*, Michael T. Brett[2☯]*, Gordon W. Holtgrieve[3], Eric J. Ward[4], Ashley P. Ballantyne[5], Carolyn W. Burns[6], Martin J. Kainz[7], Doerthe C. Müller-Navarra[8], Jonas Persson[9], Joseph L. Ravet[2], Ursula Strandberg[10], Sami J. Taipale[11], Gunnel Alhgren[12]

1 Oregon Institute of Marine Biology, University of Oregon, Charleston, Oregon, 97420, United States of America, **2** Department of Civil and Environmental Engineering, University of Washington, Seattle, Washington, 98195, United States of America, **3** School of Aquatic and Fishery Science, University of Washington, Seattle, Washington, 98195, United States of America, **4** Conservation Biology Division, Northwest Fisheries Science Center, National Marine Fisheries Service, National Oceanic and Atmospheric Administration, Seattle, Washington, 98112, United States of America, **5** Department of Ecosystem and Conservation Science, University of Montana, Missoula, Montana 59812, United States of America, **6** Department of Zoology, University of Otago, PO Box 56, Dunedin, 9054, New Zealand, **7** WasserCluster - Biological Station Lunz, Dr. Carl Kupelwieser Prom. 5, A-3293 Lunz am See, Austria, **8** Aquatic Ecology, University of Hamburg, Ohnhorststraße 18, Hamburg, D-22609, Germany, **9** Integrated Water Resources Management, Norwegian Institute for Water Research (NIVA), Gaustadallen 21, 0349, Oslo, Norway, **10** Department of Biology, University of Eastern Finland, Box 111, 80101, Joensuu, Finland, **11** Department of Biological and Environmental Science, University of Jyväskylä, PL 35 (YA), 40014, Jyväskylä, Finland, **12** Limnology/Department of Ecology and Genetics, Uppsala University, Norbyvägen 18 D, SE-75236, Uppsala, Sweden

☯ These authors contributed equally to this work.
* aaron.galloway@gmail.com (AWEG); mtbrett@uw.edu (MTB)

OPEN ACCESS

Citation: Galloway AWE, Brett MT, Holtgrieve GW, Ward EJ, Ballantyne AP, Burns CW, et al. (2015) A Fatty Acid Based Bayesian Approach for Inferring Diet in Aquatic Consumers. PLoS ONE 10(6): e0129723. doi:10.1371/journal.pone.0129723

Editor: Douglas Andrew Campbell, Mount Allison University, CANADA

Received: January 7, 2015

Accepted: May 12, 2015

Published: June 26, 2015

Copyright: This is an open access article, free of all copyright, and may be freely reproduced, distributed, transmitted, modified, built upon, or otherwise used by anyone for any lawful purpose. The work is made available under the Creative Commons CC0 public domain dedication.

Data Availability Statement: All relevant data are within the paper and its Supporting Information files.

Funding: This work was supported by the National Science Foundation grants 0925718 and 0742559 to AWEG. The funders had no role in study design, data collection and analysis, decision to publish, or preparation of the manuscript.

Competing Interests: The authors have declared that no competing interests exist.

Abstract

We modified the stable isotope mixing model MixSIR to infer primary producer contributions to consumer diets based on their fatty acid composition. To parameterize the algorithm, we generated a 'consumer-resource library' of FA signatures of *Daphnia* fed different algal diets, using 34 feeding trials representing diverse phytoplankton lineages. This library corresponds to the resource or producer file in classic Bayesian mixing models such as MixSIR or SIAR. Because this library is based on the FA profiles of zooplankton consuming known diets, and not the FA profiles of algae directly, trophic modification of consumer lipids is directly accounted for. To test the model, we simulated hypothetical *Daphnia* comprised of 80% diatoms, 10% green algae, and 10% cryptophytes and compared the FA signatures of these known pseudo-mixtures to outputs generated by the mixing model. The algorithm inferred these simulated consumers were comprised of 82% (63-92%) [median (2.5th to 97.5th percentile credible interval)] diatoms, 11% (4-22%) green algae, and 6% (0-25%) cryptophytes. We used the same model with published phytoplankton stable isotope (SI) data for $\delta^{13}C$ and $\delta^{15}N$ to examine how a SI based approach resolved a similar scenario. With SI, the algorithm inferred that the simulated consumer assimilated 52% (4-91%) diatoms, 23% (1-78%) green algae, and 18% (1-73%) cyanobacteria. The accuracy and precision of SI based estimates was extremely sensitive to both resource and consumer uncertainty, as well as the trophic fractionation assumption. These results indicate that

when using only two tracers with substantial uncertainty for the putative resources, as is often the case in this class of analyses, the underdetermined constraint in consumer-resource SI analyses may be intractable. The FA based approach alleviated the underdetermined constraint because many more FA biomarkers were utilized (n < 20), different primary producers (e.g., diatoms, green algae, and cryptophytes) have very characteristic FA compositions, and the FA profiles of many aquatic primary consumers are strongly influenced by their diets.

Introduction

Deciphering the biochemical basis of upper trophic level production is one of the key challenges in aquatic food web ecology. Different basal resources (*e.g.*, algae, aquatic bacteria, and terrestrial matter) have widely varying ingestibility, digestibility, and biochemical composition [1], and consumers (*e.g.*, herbivores, carnivores, etc.) can differ greatly in their food selectivity [2–4]. For some animals, diet can be directly assessed by stomach content analyses. However, this approach is often destructive for larger organisms, *e.g.*, marine mammals, and is not feasible for smaller organisms. Stomach content analyses may also be biased towards indigestible remnants of prey and under-represent easily digested resources [5]. For example, if a juvenile salmonid consumed equal parts of insect larvae and salmon eggs, remnants of the more difficult to digest benthic invertebrates would be more evident in partially digested stomach contents. Stomach contents are also biased towards recently consumed diets, and not what was actually assimilated over longer time periods.

Stable isotope (SI) ratios have become the main method by which trophic interactions and energetic pathways have been inferred in aquatic ecosystems [6,7]. However, only a few SI (*e.g.*, ^{2}H, ^{13}C, ^{15}N, and ^{34}S) can be applied to common ecological analyses, and the large majority of food web studies only consider isotopes of carbon and nitrogen. Carbon is useful in discriminating different types of plant diets, and nearshore versus offshore habitat use in marine environments; nitrogen is useful in helping identify trophic position. In many cases there are too many potential resources (e.g. prey) and not enough isotope tracers, which results in a mathematically "underdetermined" problem [8, 9]. For example, if only the $\delta^{13}C$ and $\delta^{15}N$ ratios are quantified, scenarios with three or less resources can be mathematically resolved, and scenarios with four or more cannot. In recent years, algorithms have been developed that can potentially deal with underdetermined problems [9], including several Bayesian based approaches [7,10,11]. Although there is much discussion in the literature regarding the underdetermined constraint, there is not a consensus as to whether the currently available data processing tools have actually resolved this problem [8, 12–14]. A logical advancement in the field would be to increase the number of source-specific biomarkers used in mixing model analyses [8].

It has long been known that the lipid composition of some marine and freshwater consumers qualitatively resembles their resources [5]. The fatty acid (FA) composition of freshwater *Daphnia* appears to be particularly strongly influenced by diet on the basis of controlled feeding experiments [15–17]. Multivariate FA signatures also vary strongly from one basal resource type to another. For example, the FA profiles of different phytoplankton and macroalgae phyla are very distinct from each other [18–20]. Dietary FAs are not strictly conservative during trophic transfer from producers to consumers. For example, some organisms have the capacity to convert 18 carbon chain (C_{18}) polyunsaturated FA (*e.g.*, α-linolenic acid) into physiologically active C_{20} or C_{22} highly unsaturated fatty acids (*e.g.*, eicosapentaenoic and docosahexaenoic

acid, respectively) [17,21]. Certain FA are selectively retained for structural, physiological or anabolic purposes, whereas others are preferentially catabolized to meet energetic demands [17]. The overall difference in the lipid profiles of consumers and their known diets can be characterized as lipid trophic modification. However, the problem of biochemical alteration is not unique to FA as isotopic trophic modification, commonly referred to in this literature as fractionation, also occurs with stable isotopes, especially nitrogen. Thus it is critical that this trophic modification is accounted for by applying "trophic enrichment factors" (TEFs) [7], directly measured in feeding trials, to each variable used in a mixing model.

Fatty acid based approaches for inferring diet have the advantage of utilizing many more potentially source-specific tracers (*i.e.*, >20), potentially resolving the underdetermined constraint identified for SI-based mixing models [8–9,12–14]. The Bayesian mixing model approach is a powerful framework for estimating consumer diets using FA because the existing models, which can be easily adapted for new variables [22–24], are designed to both account for uncertainty and characterize the distribution of the most likely mixing model solutions, which differs considerably from earlier quantitative modeling methods involving FA, *e.g.*, Iverson et al. [25]. The probabilistic picture of the solutions obtained with the Bayesian approach shows the shape and uniformity of the solutions, and is therefore more informative than the confidence interval alone obtained with other methods.

We developed and validated a quantitative framework for the use of FA biomarkers (*e.g.*, >20) to generate robust inferences of biochemical and energetic pathways in aquatic food webs. We explicitly tested the hypothesis that using 20+ dietary tracers would give more accurate and precise outcomes than using 2 tracers in a Bayesian mixing model framework [8,13]. The additional objectives of this study were: 1) to determine if consumer FA composition could be used quantitatively to infer diet using the Bayesian mixing model framework previously developed for SI [7,10,11]. We used the FA profiles of *Daphnia* spp., which had consumed a wide variety of phytoplankton taxa [1,15,16] in 34 controlled laboratory experiments, to build a comprehensive 'consumer-resource library' of the signatures of *Daphnia* fed these resources. 2) We simulated *Daphnia* FA and SI profiles consisting of mixtures of phytoplankton diets and analyzed the theoretical *Daphnia* signatures with a modification of MixSIR [10], i.e., the Fatty Acid Source-Tracking Algorithm in R (FASTAR). 3) Using phytoplankton SI and FA data, and assuming known fractionation, we compared the relative performance of SI and FA based approaches for determining which algal groups contributed to *Daphnia* biomass when consuming mixed phytoplankton assemblages.

Methods

We conducted 34 feeding trials where *Daphnia magna* or *Daphnia pulex* were fed *ad libitum* diatom (Bacillariophyceae; n = 10), green algae (Chlorophyceae; n = 8), cryptophyte (Cryptophyceae; n = 8) or cyanobacteria (n = 8) monocultures. The FA composition of the *Daphnia* and their diets were determined in each case. The results of these experiments have for the most part been previously reported [1,15,16], but all of these samples were rerun to standardize their chromatography. Fatty acid methyl esters (FAME) were analyzed with a gas chromatograph (HP 6890) equipped with a flame ionization detector, separated using an Agilent DB-23 column (30 m length, 0.25 mm diameter, 0.15 μm film thickness), and identified with a FA standard mixture (37-component FAME, Supelco, Bellefonte PA), and mass spectrometry for FAME not included in the standard [20]. The FA profiles (n = 26 FA) of all *Daphnia* in the consumer-resource library, as well as the pseudo-*Daphnia* profiles created for the tests, were visualized as a multivariate 'resource polygon' with non-metric multidimensional scaling (NMDS) and Euclidean distance (metaMDS and ordihull functions with Vegan library in R).

In order to compare against phytoplankton phyla-level FA based solutions, we also generated a comparable data set for phyla level differences in carbon and nitrogen SI ratios. As far as we are aware, Vuorio et al. [26] are the only researchers to report detailed and empirically derived determinations of phytoplankton SI ratios for multiple phyla and lakes, because of the difficulty of separating specific phytoplankton from seston/particulate organic matter. Phytoplankton SI ratios from laboratory studies are not applicable because they primarily depend on the SI ratios of the nitrate and bicarbonate used in the algal growth media. We calculated the mean ± SD for isotopic ratios for cyanobacteria, chlorophytes and diatoms for each lake reported in Vuorio et al. [26]. Conceptually the mean SI values were obtained from the group specific lake means, and the uncertainty values were obtained from the within lake group specific SD values. To obtain uncertainty estimates that only reflect within lake variation, the between lake variability in these data was removed by first calculating the average SI ratios (± SD) for cyanobacteria, because cyanobacteria are the phylum with the most data. Cyanobacteria had a mean δ^{13}C value of -25.8‰ across lakes, and an average variability of ± 1 SD = 3.0‰ within lakes. The normalized δ^{15}N values for cyanobacteria were similarly found to be 2.8 ± 2.5‰ (Table 1). Diatom δ^{13}C ratios were on average 4.0 ± 3.3‰ depleted compared to cyanobacteria and these ratios varied by ± 1.3‰ within lakes, which gave a pooled within lake diatom δ^{13}C estimate of -29.8 ± 2.3‰. Similarly, a diatom δ^{15}N estimate of 7.5 ± 1.5‰ was obtained (Table 1). Green algae were only sampled from one lake [26], where their δ^{13}C ratios were 2.0‰ enriched relative to cyanobacteria. Because of this small sample size, the pooled uncertainty for cyanobacteria and diatoms (*i.e.*, ± 2.6‰) was used to represent δ^{13}C uncertainty for green algae. This provided a δ^{13}C value of -23.7 ± 2.6‰ for green algae. Using the same approach, a δ^{15}N value of 8.7 ± 2.0‰ was obtained for this group (Table 1).

To assess if FA biomarkers can be used to quantitatively estimate diet proportions, and how these estimates compare to those inferred from SI data, we applied the MixSIR model of Moore and Semmens [10] to both FA and SI data. The FASTAR algorithm is essentially identical to MixSIR in its statistical approach (e.g., the likelihood, calculation of variances, and assumptions about errors). Similar to MixSIR, the FA-based algorithm is configured so that is can accommodate any number of tracers, resources or consumers. FASTAR also directly accounts for consumer lipid fractionation, which is an important conceptual difference with both MixSIR and SIAR. Finally, the FA-based algorithm independently aggregates the results when multiple consumers are analyzed simultaneously. This difference means dispersion in the outputs does not change if increasing numbers of consumers are considered [14].

The equation that describes the relative abundance of individual FA (or SI ratios) in a consumer as a result of mixing from multiple dietary sources with variable signatures is:

$$u_j = \sum_{i=1}^{n} p_i (m_{j,i} + f_{j,i})$$

where n represents the number of sources, $m_{i,j}$ and $f_{i,j}$ represent the mean and fractionation of source item i with respect to stable isotope or fatty acid j (often obtained in laboratory settings),

Table 1. The normalized phytoplankton SI values from Vourio et al. [26].

	δ^{13}C	δ^{15}N
Diatoms	-29.8 ± 2.3‰	7.5 ± 1.5‰
Chlorophytes	-23.7 ± 2.6‰	8.7 ± 2.0‰
Cyanobacteria	-25.8 ± 3.0‰	2.8 ± 2.5‰

These values reflect within lake variation in SI values for the major phytoplankton groups. The reported values are the mean ± SD.

doi:10.1371/journal.pone.0129723.t001

the vector $\underline{p}$ represents the estimated proportions or relative contributions for each source to the consumer (constrained to sum to 1.0), and u_j represents the mean of the mixture, as a derived parameter. The mixture variance can also be expressed in terms of the variances of the sources (s_{ij}^2) and fractionation (g_{ij}^2),

$$\sigma_j^2 = \sum_{i=1}^{n} p_i^2 (s_{j,i}^2 + g_{j,i}^2)$$

[10].

Because our libraries of dietary source data are based on *Daphnia* feeding trials, which directly incorporate modification of dietary signatures by the consumer, we did not include trophic fractionation in the FA mixing model.

A critical assumption of mixing models is that the likelihood is specified correctly. For Bayesian mixing models like MixSIR or SIAR, the mixture mean (and consumer data) are assumed to be normally distributed (via the Central Limit Theorem). One difference in applying the mixing model with FA data compared to SI data is that SI measurements for sources (e.g. prey signatures) are approximately normally distributed, whereas for FA data, the source data are proportions (constrained between 0 and 1). For models like MixSIR or SIAR, the distribution of the mixture is normally distributed because the source means are normally distributed, and the mixture is a linear function of the source means and estimated proportions [10]. What makes the analysis of FA data different is that FA signatures are usually reported as proportions, and thus the distribution of a single fatty acid may not be normal. When the signatures from multiple FA are combined with the estimated proportions (source contributions), however, their sum is approximately normally distributed. Further, this approximation improves as more FAs are included. We also used a z-score transformation for all of the FA that averaged $>$ 5% of total FA to directly test whether the dominant FA were or were not normally distributed.

Following the analysis of SI data in Bayesian mixing models, we analyzed FA data using the same framework to allow the option of adding prior information and to provide posterior probability distributions that describe the full range of likely proportional contributions from potential prey given the data and our model. For this analysis we assumed the Dirichlet distribution ($\alpha = 1$) prior in compositional space. The Dirichlet with $\alpha = 1$ is uniform on the combinations of proportions, but is not necessarily uniform for single proportions. We also tested an alternative prior with the α as uniform hyperparameters, ~ Uniform(0,100). Alternative priors, such as the Jeffreys' prior ($\alpha = 0.5$) are also feasible, however a full comparison of alternative priors is beyond the scope of this analysis. It should also be noted that the vast majority Bayesian mixing model analyses that utilize stable isotopes use the Dirichlet prior [7]. The posterior distributions were estimated using the Gibbs sampling algorithm of Markov Chain Monte Carlo (MCMC) implemented using the open source Just Another Gibbs Sampler (JAGS) software [27] via the R statistical software environment [28]. MCMC chains were run for 100,000 iterations with a 50,000 iteration burn-in and a thinning rate of 50. The model was run individually among replicate consumers, each with their own set of posterior results; therefore, the posterior distribution for the set of replicate consumers is the sum of the individual posteriors. Separating individual consumers essentially treats each as a fixed effect; alternative approaches for analysis would be to combine all consumers in the same analysis assuming the same shared diet [7,10] or to treat individuals as random effects and estimate the deviation of each from a global mean [29]. Treating each individual separately also does not cause dispersion to collapse when analyzing multiple consumers simultaneously [14], which does occur when using the conventional scripts for MixSIR and SIAR [7,10]. The code and dependent data files used for these simulations are provided in S1 File.

To initially test the algorithm, we ran the 26 cases where *Daphnia* consumed diatom, green algae, or cryptophyte monocultures through the algorithm to test whether this code would correctly classify the primary data on which it was based. For example, *Daphnia* that solely consumed *Cyclotella*, *Scenedesmus* or *Rhodomonas* were analyzed. We then used a Monte Carlo approach and the distributions of diatom, green algae and cyanobacteria SI values, to generate 1000 realizations in groups of n = 100 of simulated *Daphnia* that were comprised of 100% diatoms, 100% green algae or 100% cyanobacteria. These cases were processed using the same code as above. These hypothetical cases included the full uncertainty associated with both the resource and isotopic fractionation. For example, in the case of pure diatom diets, the 1000 cases tested had a mean ± SD value of -29.8 ± 2.7‰ and 7.5 ± 1.8‰ for $\delta^{13}C$ and $\delta^{15}N$, respectively.

To test whether the algorithm could resolve mixed diets, we used the initial dataset where *Daphnia* had consumed diatoms, green algae or cryptophytes to generate "pseudo-*Daphnia*" that were comprised of 80% diatoms, 10% green algae, and 10% cryptophytes. This 80/10/10 scenario was chosen so that it would be clearly different from the generalist prior (i.e., 33.3/33.3/33.3). The FA profiles for *Daphnia* consuming each individual phytoplankton group were randomly combined (*e.g*., diatom #4, chlorophyte #7, cryptophyte #2) by multiplying the *Daphnia*-diatom profile by a weight of 0.8, the *Daphnia*-green algae profile by 0.1, and the *Daphnia*-cryptophyte profile by 0.1, and summing these values into a single hypothetical profile. This process was repeated with random resampling 1000 times, with hypothetical consumers processed in groups of n = 100.

Similarly, we used the SI data and a Monte Carlo approach to generate 1000 *Daphnia* realizations that had consumed 80% diatoms, 10% green algae and 10% cyanobacteria. These pseudo-*Daphnia* reflected the full uncertainty associated with their food sources. These hypothetical consumers also accounted for the uncertainty associated with trophic fractionation, *i.e*., ± 1.3‰ and ± 1.0‰ for the $\delta^{13}C$ and $\delta^{15}N$ ratios, respectively [30]. This resulted in pseudo-*Daphnia* with mean $\delta^{13}C$ and $\delta^{15}N$ ratios of -28.7 ± 2.3‰ and 6.9 ± 1.6‰, respectively.

To explore which sources of uncertainty most affected the outputs of the FA and SI based analyses, these analyses were repeated after minimizing the uncertainty associated with the putative resources as well as the *Daphnia* in a 2 by 2 matrix (see below). Because the MixSIR framework does not allow resources to have zero uncertainty (variance in consumers being a weighted mixture of variance from sources), the SD values for the consumer-resource library file were divided by 100 to create scenarios with virtually no uncertainty. The pseudo-*Daphnia* biomarker signatures for this scenario were created to be the result of perfect composition of 80% diatoms, 10% green algae, and 10% cryptophyte, with zero uncertainty (*i.e*., SD = 0). This resulted in four cases: 1) resource uncertainty and consumer uncertainty = 100%, 2) resource uncertainty = 100% and consumer uncertainty = 0%, 3) resource uncertainty ≈ 0% and consumer uncertainty = 100%, and 4) resource uncertainty and consumer uncertainty ≈ 0%.

We also tested the importance of the fractionation uncertainty for SI based calculations by setting the resource and consumer uncertainty equal to zero and varying the fractionation assumption for C and N by fixed values of ± 1.96 SD to represent a 95% confidence interval. This resulted in a 2 by 2 matrix of outcomes, and provided a quantitative measure of how much typical SI based outputs can vary due to uncertainty for consumer stable isotope fractionation.

Results

Dietary effects on consumer lipid composition

The phytoplankton and *Daphnia* data compiled for this study show different phytoplankton groups have very distinct FA composition and the FA profiles of *Daphnia* are strongly influenced by their diets (Fig 1, also see Supporting Information, S1 Table). *Daphnia* that consumed

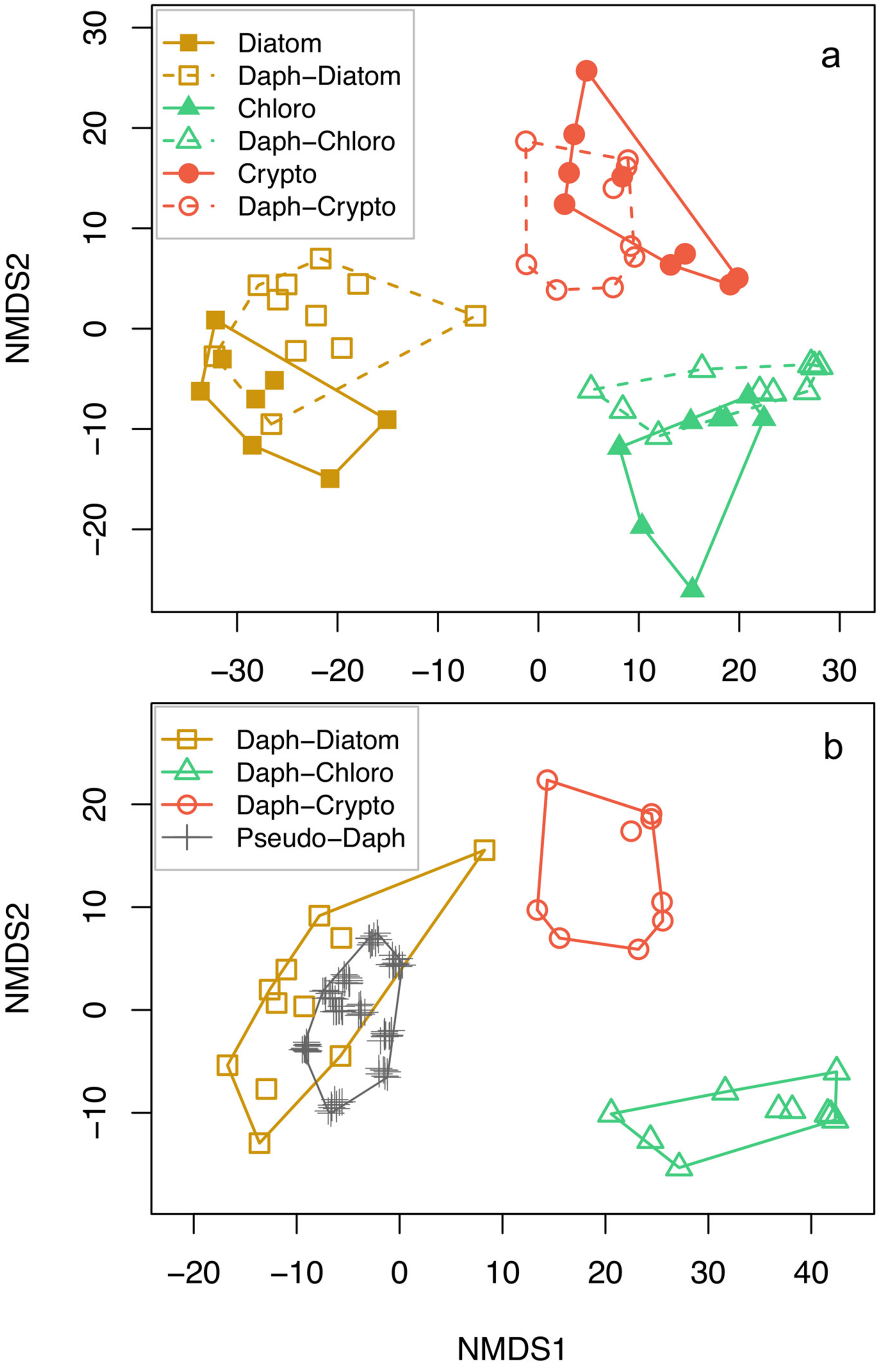

Fig 1. Non-metric multi-dimensional scaling (NMDS) plots of the FA profiles used (n = 26 FA; Euclidean distance). One outlier cryptophyte profile is removed from the NMDS plots for clarity, but this outlier was included in the FA-based analyses. A) Algal diets (filled symbols) and *Daphnia* fed those diets (open symbols) and associated resource polygons for each treatment group (stress = 0.09). B) The real *Daphnia* in the 'consumer-resource library' (no algal diets), with 100 'pseudo-*Daphnia*' used in the analyses (see Methods; stress = 0.07).

doi:10.1371/journal.pone.0129723.g001

diatoms, green algae and cryptophytes had FA profiles that were strongly statistically associated with their diets (*i.e.*, r^2 = 0.87 ± 0.04), but weakly associated with conspecifics consuming alternative diets (*i.e.*, r^2 = 0.19 ± 0.18). The diatom diets used in our experiments were characterized by high proportions of the FAs 14:0, 16:2ω7, 16:3ω4, 20:5ω3, and especially 16:1ω7. Furthermore, *Daphnia* that consumed diatoms were characterized by high proportions of these same fatty acids. Green algae, and the *Daphnia* that consumed green algae, were characterized by high proportions of 16:2ω6, 16:3ω3, 16:4ω3, and especially 18:1ω9, 18:2ω6 and 18:3ω3. Both cryptophytes, and the *Daphnia* that consumed cryptophytes, were enriched with 18:3ω3, 18:4ω3 and 20:5ω3. Despite these similarities, *Daphnia* generally had more 20:4ω6 and 20:5ω3, and less 22:5ω6 and 22:6ω3 than their algal diets.

Priors

We tested two different prior functions to explore how these affected model outputs, *e.g.*, the standard default uniform Dirichlet distribution (α = 1) for SIAR [7] as well as the alternative prior dunif(0,100) on the alphas. These priors were tested for an 80/10/10 scenario using the same exact datasets for SI and FA based analyses using both MixSIR and SIAR resulting in eight different outcomes (S2 Table). For FA based analyses, the two priors gave similar mean and median outcomes, but the 95% credibility intervals were slightly larger for the alternative prior. For SI based analyses, the Dirichlet prior gave more accurate outcomes by about 2–3% in absolute terms albeit with larger credibility intervals (S2 Table).

Z-score distribution

An analysis of the normalized z-scores for the fatty acid values used to generate our consumer-resource library showed these data were approximately normally distributed (S1 Fig). The standard deviation for these data was 0.95, and the median (-0.15) was only 4 percentiles skewed relative to the mean.

100% diet contribution scenarios

For the 26 cases where *Daphnia* were fed algal monoculture diets, *i.e.*, diatoms (n = 10), green algae (n = 8) and cryptophytes (n = 8), the FA profiles for the individual cases had strong statistical associations with the averages for their respective resource libraries (*i.e.*, the r^2 = 0.88 ± 0.07). For these 26 cases, the algorithm in all cases correctly classified the diet contributions from the different algal groups to multiple decimal places (Fig 2). The algorithm was much less capable of classifying diet using carbon and nitrogen SI. The three scenarios tested (*i.e.*, 100% diatoms, 100% green algae and 100% cyanobacteria), generated nine outcomes (*i.e.*, three for each scenario). The actual outcomes for the three 100% cases, and the six 0% cases, were very similar within group so the results of these groups were pooled into two sets of responses. For the three cases that should have had a 100% contribution, the algorithm median and 95% credible interval (*i.e.*, the 2.5th to 97.5th percentile range of the model solution posterior density) contribution was 64% (3–95%) (Fig 2). In the six cases that should have had a 0% contribution, the modeled median contribution was 14% (0–80%). In the 100% cases, the posterior distribution was very flat and few outcomes were excluded from the 95% credible interval (Fig 2), and the correct answer was one of the few outcomes that was excluded. In both the 100% and 0% cases, the output values were very similar to the average of the prior expectation (*i.e.*, equal contributions from the three putative resources) and correct values, *i.e.*, 67 and 17%, respectively.

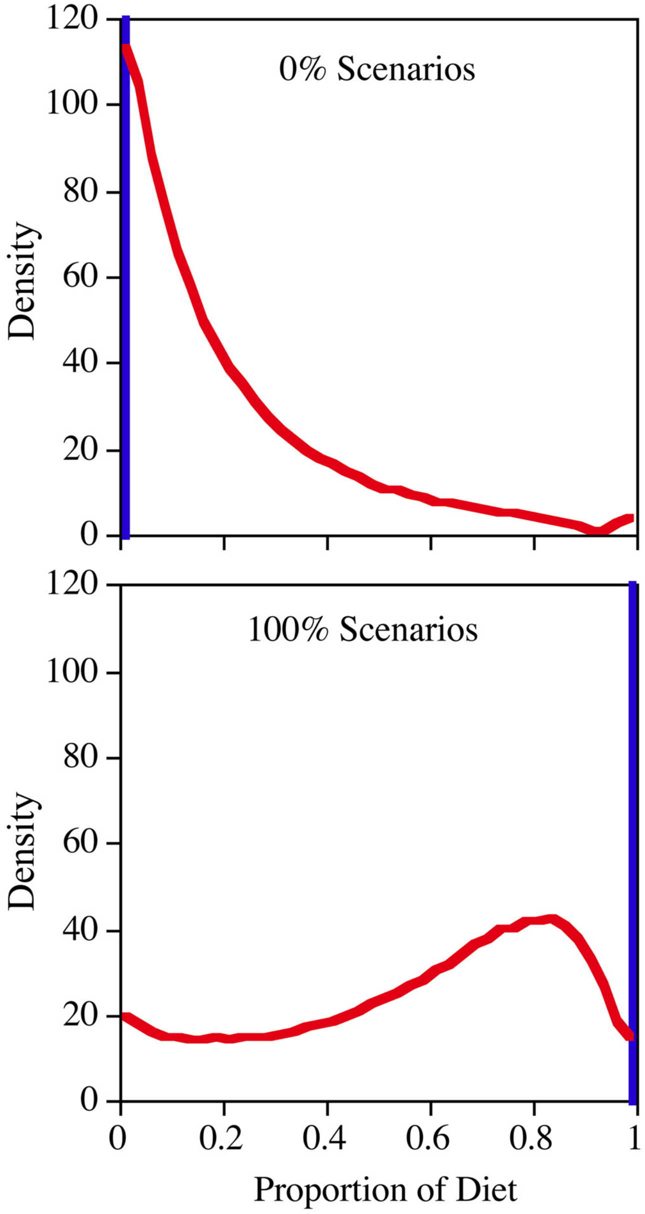

Fig 2. The results of an analysis where three potential food resources were either set to 100% of the food consumed or 0% (*e.g.*, 100% diatoms, 0% green algae, and 0% cyanobacteria). This resulted in 9 outputs for the three SI based analyses (red). Because the outputs for the three SI cases where the subsidy should have been 100% were very similar, as was also true for the six cases where the subsidy should have been 0%, the three 100% responses and the six 0% responses were aggregated in this plot. For the FA based analyses (blue), we simply analyzed the original 10 cases where *Daphnia* consumed diatom monocultures. We also analyzed the 8 cases where the *Daphnia* consumed green algae monocultures as well as the 8 cases where they consumed cryptophyte monocultures. In these 26 cases, the correct answer was always obtained to multiple decimal places. The curves represent the 1/10th percentile (n = 1000) density distribution of the model posterior densities grouped into 40 bins.

doi:10.1371/journal.pone.0129723.g002

Mixed diet contribution scenarios

The 80% diatom, 10% green algae, and 10% cryptophyte mixture scenarios showed the FA-based algorithm was reasonably effective at providing both an accurate and precise answer when hypothetical mixed diets based on lipid profiles were analyzed (Fig 3). For example, the median and 95% credible intervals were 82% (63–92%) for diatoms, 11% (4–22%) for green algae, and 6% (0–25%) for cryptophytes.

The SI based analyses of a similar 80% diatoms, 10% green algae, and 10% cyanobacteria *Daphnia* scenario produced much less accurate and much more variable results (Fig 3), which were again nearly exactly intermediate between the prior assumption and the actual composition of the pseudo-*Daphnia*. In this case, the algorithm inferred the pseudo-*Daphnia* consumed 52% (4–91%) diatoms, 23% (1–78%) green algae, and 18% (1–73%) cyanobacteria. The inferred diatom contribution was incorrect by 28% in absolute terms, and the 95% uncertainty interval (87%) was similar to that of a completely flat posterior distribution. The only potentially useful and accurate output from the SI based analyses was the exclusion of very large dietary contributions from green algae and cyanobacteria.

Resource and consumer uncertainty

The sensitivity analyses of resource and consumer uncertainty contributions to model error for the FA based analyses showed consumer uncertainty caused somewhat better precision than did resource uncertainty. For example, the calculated contribution from diatoms was 81% (70–88%) when the resource uncertainty was ≈ 0% and consumer uncertainty = 100%. By comparison, when the resource uncertainty was set to 100% and consumer uncertainty was set to 0%, the diatom contribution was calculated to be 82% (66–90%). In this case, accuracy and precision were also slightly worse for the green algae and cryptophytes. When both the resource and consumer uncertainty were minimized, the algorithm was extremely accurate for diatoms, *i.e.*, 80.00% (79.7–80.3%), as well as for green algae and cryptophytes.

Uncertainty arising from resources was somewhat less important for SI based analyses than that arising from consumers. When the resource uncertainty was set to 100% and consumer uncertainty was set = 0%, the calculated contributions for diatoms were very inaccurate, *i.e.*, 56% (6–90%), and were only slightly better than when both the consumer and resource uncertainty were set equal to 100%. When the resource uncertainty was minimized, and the consumer uncertainty was kept at 100%, output accuracy and precision declined, *i.e.*, the diatom contribution was calculated to be 46% (2–90%). However, when both resource and consumer uncertainty were minimized, the SI based analysis also gave a very accurate outcome for the diatom contribution, *i.e.*, 80% (78–82%). Similar results were obtained for the green algae and cyanobacteria outcomes in all four cases.

Fractionation uncertainty

Sensitivity analyses examining uncertainty due to consumer fractionation showed varying this assumption by ± 1.96 SDs for both carbon and nitrogen, with no resource and consumer uncertainty, dramatically affected MixSIR outputs and resulted in a range of median outputs that varied by 55–81% in absolute terms (Table 2).

Discussion

We have shown that a fatty acid biomarker approach within a Bayesian mixing model framework [10,11] can be used to infer consumer diets with accurate and fairly precise outcomes. This approach provided much better results than did a traditional SI approach using two

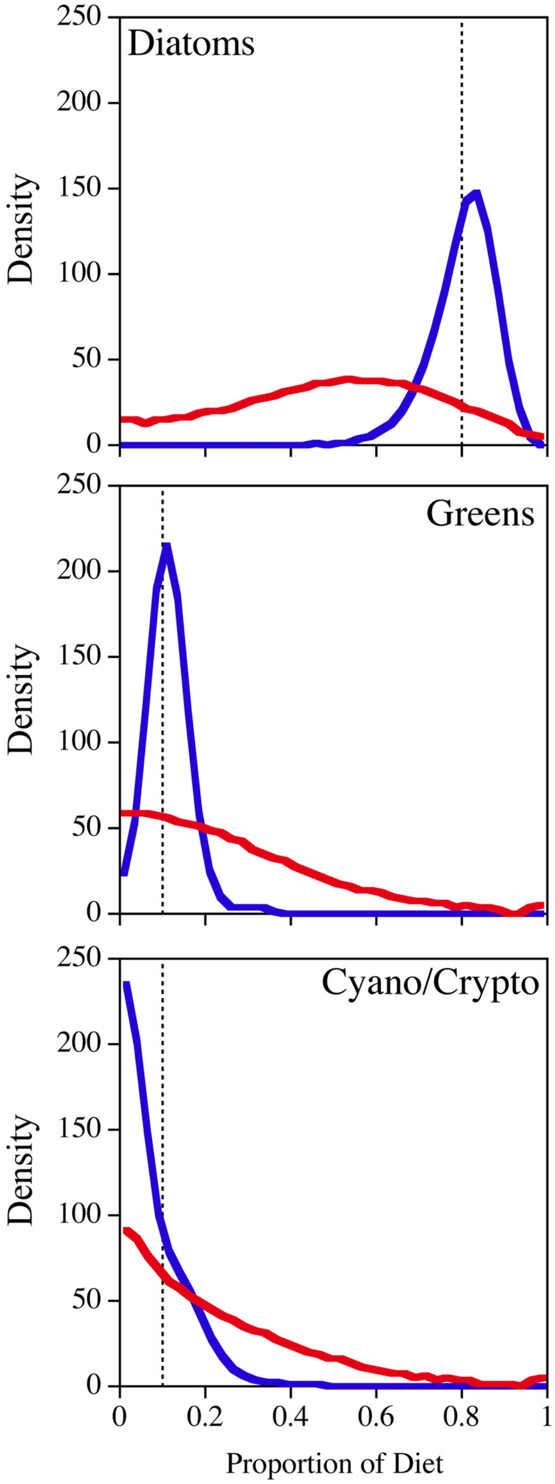

Fig 3. Results from mixed diet simulations for "pseudo-*Daphnia*" that were comprised of 80% diatoms, 10% green algae and 10% either cyanobacteria or cryptophytes for the SI (red) and FA (blue) based analyses. These scenarios included full uncertainty for both the resources and fractionation (n = 1000, in groups of 100). The density curves represent the posterior distributions of the 1/10th percentiles (n = 1000) grouped into 40 bins.

doi:10.1371/journal.pone.0129723.g003

Table 2. A sensitivity analysis of the influence of the carbon and nitrogen fractionation assumptions on MixSIR SI based outputs.

	Median	Mean ± SD
Carbon (mean), Nitrogen (mean)		
Diatoms	0.80	0.80 ± 0.01
Greens	0.10	0.10 ± 0.01
Cyanos	0.10	0.10 ± 0.01
Carbon (+1.96 SD), Nitrogen (+1.96 SD)		
Diatoms	0.30	0.30 ± 0.01
Greens	0.70	0.70 ± 0.01
Cyanos	0.00	0.00 ± 0.00
Carbon (+1.96 SD), Nitrogen (-1.96 SD)		
Diatoms	0.19	0.19 ± 0.01
Greens	0.25	0.25 ± 0.01
Cyanos	0.55	0.55 ± 0.01
Carbon (-1.96 SD), Nitrogen (+1.96 SD)		
Diatoms	1.00	0.86 ± 0.35
Greens	0.00	0.14 ± 0.35
Cyanos	0.00	0.00 ± 0.00
Carbon (-1.96 SD), Nitrogen (-1.96 SD)		
Diatoms	0.84	0.84 ± 0.01
Greens	0.00	0.00 ± 0.00
Cyanos	0.16	0.16 ± 0.01

In these scenarios, resource and consumer uncertainty was minimized and consumer fractionation was varied by ± 1.96 SD. These results show the influence of various fractionation assumptions on the outputs in the absence of other sources of uncertainty.

doi:10.1371/journal.pone.0129723.t002

tracers ($\delta^{13}C$ and $\delta^{15}N$). The superior performance of the FA based approach is due to the fact that many more biomarkers can be used resulting in more degrees of freedom to constrain the plant-herbivore interface, and many of these biomarkers are strongly source specific. An important and novel aspect of the FA based approach used here is that it explicitly accounts for consumer trophic modification of FA by basing its outputs on a 'consumer-resource library', *i.e.*, a compilation of FA profiles of consumers fed defined diets (Fig 1). We therefore did not need to correct the pure algal FA signatures with generalized 'calibration coefficients' [25] or trophic enrichment factors (TEFs) [7]. TEFs are well known to strongly influence the outcome of mixing model analyses [31,32], as we also observed in our SI fractionation sensitivity analyses.

Our results validate the prediction that using additional tracers will result in more accurate, source-specific information, and more precise Bayesian mixing model outcomes. The fact that 20+ tracers give more accurate and precise results than 2 tracers may seem self-evident, however, the vast majority of food web stable isotope analyses are "are multivariate with dimension 2" [33]. Our results also show that the problem inherent in two stable isotope based mixing model analyses is not the stable isotopes themselves or even the code used to process the data, but rather the small number of tracers commonly used in these types of problems and the underdetermined constraint. In fact, the MixSIR model with 2 tracers had excellent performance provided only three resources were considered and these resources had distinct stable isotope ratios. Recently, many studies have adopted δ^2H as third tracer or even as a replacement tracer for $\delta^{15}N$ in two stable isotope analyses [34]. However, including δ^2H values does

not alleviate the underdetermined constraint in three isotope applications because it introduces two unknowns, *i.e.*, the contribution to the consumer and the SI signature from dietary water, and the latter term is highly uncertain [34]. In two isotope applications replacing $\delta^{15}N$ with $\delta^{2}H$ exacerbates the underlying algebraic constraint because of the additional unknown. Moreover, assuming the fractionation assumptions are met, a three-isotope model is still mathematically limited to resolving a four-source system [8,13]. Given these results for FA and SI data, one avenue for future research is to determine what number of tracers yields the best inference and model performance?

Our results indicate FA based TEFs are likely to be algal group specific. This is not a limitation that is unique to FA biomarkers; substantial variations in TEFs are well known to exist for SI [32,35]. Therefore, we advocate for the general approach used here, where trophic fractionation is measured with feeding trials and accounted for natively within the model because it ensures that biomarker and diet-specific fractionation is properly accounted for within a given organism. It would have been possible to calculate TEFs based upon our experimental data and include these diet- and FA-specific TEFs in the model with a fractionation file [10], however, this would produce equivalent results, and might encourage future users to erroneously apply the *Daphnia*-specific TEFs found in our study to other organisms without testing. Although the consumer-resource library is data intensive, it is a critical feature of the FASTAR approach. This is because consumers FA profiles will also always exhibit some systematic differences relative to their diets (S1 Table), even though their lipid profiles are very strongly influenced by their diet [15,16]. For example, Taipale et al. [17] found *Daphnia* had less saturated FA, and more 20:5ω3 and 20:4ω6, than their diets. Strandberg et al. [36] found *Daphnia* converted nearly all of the dietary 22:5ω6 to 20:4ω6. It is also likely that most of the 22:6ω3 consumed by *Daphnia*, which cladocera do not accumulate [15,37], will be similarly retroconverted to 20:5ω3.

Studies on consumer SI fractionation indicate fractionation can be dependent on diet, consumer type and the physiological state of the consumer [30,32,35], and averages 0.4 ± 1.4‰ for $\delta^{13}C$ and 3.4 ± 1.0‰ for $\delta^{15}N$ [30]. The conventional practice when analyzing SI of field-collected organisms is to simply assume the global average fractionation value for all consumers. However, this approach is logically flawed and can introduce quite substantial error in model outputs. The problem with assuming the average fractionation value is that any particular consumer will, for the systematic reasons mentioned above, have its own characteristic (but unknown) fractionation values that will almost always be different from the global means for all consumers. For example, Adams and Sterner [38] found a wide range on $\delta^{15}N$ fractionation in *Daphnia* depending on the carbon to nitrogen ratio of their diets and Prado et al. [39] identified diet dependent fractionation for both $\delta^{13}C$ and $\delta^{15}N$ in experimentally raised sea urchins. When we tested the effect of different fractionation assumptions on the SI based outputs, we found strongly differing outputs depending on the combination of fractionation values that were assumed (Table 2). It is also not unusual for the fractionation uncertainty to be as large as the differences in the SI ratios for the putative food resources [40]. The fact that the FA based method for inferring diets requires direct determination of lipid dietary modification in consumers could be considered to be a disadvantage because these types of feeding trials can be quite time consuming. Conversely, these feeding trials directly resolve the fractionation quandary so this source of uncertainty is largely eliminated from the mixing model calculations. As more feeding trials are completed, it may be possible to make generalized assumptions regarding lipid fractionation within particular consumer groups. However, since different consumer groups (e.g., cladocerans and copepods) have distinct lipid fractionation [16], generalizations should only be made for specific groups on the basis of controlled experiments.

We generated the consumer-resource library from 34 experiments where *Daphnia* were fed phytoplankton monoculture diets (Fig 1). To generate this type of a consumer FA composition data set it is essential that organisms can be grown on monoculture diets, and that the consumer's growth in these experiments is sufficiently large so that the initial maternal contribution to neonate lipids is diluted by newly acquired FA. In the case of *Daphnia* fed diets comprised of diatoms, green algae and cryptophytes, *Daphnia* neonates grew very rapidly, increasing by 50 to 100 times from their initial mass in 10 d feeding trials. However, many organisms cannot be successfully reared in laboratory studies, or grow so slowly that very long feeding trials are needed. Resource libraries may also be developed from published feeding trial data from experiments not originally designed for this purpose, particularly for slow growing species fed known diets for aquaculture related research, *e.g.*, bivalves [41]. Recently, we have also compiled an analogous consumer-resource library for the marine isopod *Idotea wosnesenskii* experimentally fed green, red, and brown marine macroalgae, and used this library to generate quantitative estimates of wild isopod resource utilization [42]. We have also applied the FASTAR algorithm to a cladocera dataset for large-sized humic lakes in Finland to estimate the relative importance of terrestrial particulate organic matter, bacterioplankton, and several phytoplankton groups to zooplankton production in these lakes [43], using an independent *Daphnia* library dataset for Finnish humic lakes. Over time, such experiments will generate robust resource libraries of FA signatures for many consumers that have been fed diverse basal resources, under various laboratory conditions, thereby increasing the potential applications to field data.

In the present study the quality of our consumer-resource library file for *Daphnia* consuming cyanobacteria was poor because *Daphnia* fed cyanobacteria grew poorly and still had features in their lipid profiles that suggested residual maternal lipids. Specifically, the maternal *Daphnia* were usually reared on the green alga *Scenedesmus* and *Daphnia* in the cyanobacteria treatments had more 18:1ω9, 18:2ω6 and 18:3ω3 than would be expected based on a cyanobacteria diet. We attempted to correct this problem by averaging the *Daphnia* FA profiles in these experiments with the original profiles for the diets to obtain consumer-resource library values that were more indicative of cyanobacteria consumption. However, at this time, the cyanobacteria data in our consumer-resource library are problematic and follow up studies will be required before this problem is resolved. Field applications of our approach should acknowledge this limitation for any outputs pertaining to cyanobacteria [43]. A combination of FA, bulk $\delta^{15}N$ [26], and compound-specific analyses ($\delta^{15}N$) of amino acids [44], would likely separate cyanobacteria from eukaryotic phytoplankton.

There are cases where a FA based mixing model methodology likely cannot be used to resolve consumer resource utilization questions, such as identifying the relative importance of benthic and pelagic resources for lake consumers [45]. In this case a FA approach would not differentiate, for example, between benthic and pelagic diatom contributions to consumer diets because these different food resources have very similar FA profiles. Similarly, in marine ecology there is considerable interest in the importance of ice-algae to polar food webs, especially as sea ice recedes dramatically in unison with climatic change [46]. In this case, the dominant primary producers in the ice-algae and phytoplankton communities, *i.e.*, pennate and centric diatoms, respectively, also have very similar FA composition. However, in both the benthic versus pelagic and ice-algae versus phytoplankton cases, the different diatom groups have quite different CO_2 sources that would also likely have distinct $\delta^{13}C$ ratios. Therefore in these cases, a conventional stable isotopic analysis or compound-specific SI analyses of particular FA might provide more insight into trophic interactions [17,36]. Fatty acid and stable isotope data can be seamlessly combined when using the FA-based algorithm. The choice of biomarkers used in the model, (*e.g.*, FA, sterols, amino acids, or SI) should be based on the ability of

potential biomarkers to clarify contributions from particular resources, as certain biomarkers will prove to be much more informative than others.

One of the key distinctions between FA and SI based mixing model applications for consumer resource subsidy applications is that the FA composition of particular primary producer groups is primarily dictated by phylogenetic relationships and to a much lesser extent environmentally controlled [20]. Temperature, nutrients and light do influence primary producer FA composition [47], but a diatom will always have a FA composition that is very distinct from, for example, green algae irrespective of environmental conditions. However, the FA plasticity of mixotrophs during various environmental conditions clearly needs further attention, as it is possible that mixotrophs may modify their FA according to their environmental requirements. Conversely, the SI composition of different primary producers is mainly a function of the available sources of, for example, CO_2 and NO_3^- in an aquatic system, and because different primary producers tend to compete for the same pool of nutrients their SI ratios will also tend to be similar spatially and temporally.

Potential sources of error for FASTAR in field applications include the mismatch between the FA profiles for the cases used to generate the consumer FA composition library and the actual phytoplankton taxa consumed in the field. For example, our consumer-resource library for *Daphnia* consuming diatoms is based on experiments conducted using *Asterionella*, *Aulacoseira*, *Cyclotella*, *Fragilaria*, *Navicula*, *Stephanodiscus*, and *Synedra*. In actual field applications zooplankton could be consuming different diatom taxa such as *Tabellaria* and *Diatoma*. However, it is unlikely that the FA profiles of these genera would be dramatically different from those already included in our library [20]. Furthermore, as this research tool is utilized more FA profiles will be generated and could be included in a global library, specific to particular consumers, that could be queried by other researchers. Both temperature and starvation have secondary influences on zooplankton FA composition independent of the primary influence from diet [48] and could therefore result in some diet misclassification. Diet misclassification will also arise if the potential food resources are mis-specified in the algorithm or if key resources are missing from the library. Further, the consumer-resource library approach implicitly assumes the zooplankton analyzed have consumed exactly equal portions of the specific taxa used in our feeding trials. This will never be the case in field systems. However, this irreducible source of error was directly accounted for in the 1,000 cases we tested for the 80/10/10 scenario. It should also be emphasized that the consumer-resource library will almost certainly be consumer specific, so if different consumers are assessed (*e.g.*, calanoid copepods, chironomids, gammarids, etc.) new resource libraries must be compiled. Finally, it is essential that the same suite of FA be used for the field samples and reference library files. For example, our reference library includes several C_{16} PUFAs, as well as 18:4ω3, which are not usually included in the fatty acid standard mixtures used to calibrate chromatography. If the current reference library was applied to field datasets where these FAs had not been quantified, a misclassification error would be introduced.

FAs are promising biomarkers because a single relatively simple analysis can yield many dietary tracers. We are hopeful that future research will identify additional dietary tracers beyond FAs and SIs that can be used for dietary inference in a Bayesian framework. A wide variety of biochemicals including FAs, amino acids, sterols, plant pigments and even elements have been identified as being important to aquatic consumers [49], but for a variety of reasons many of these are not ideal dietary biomarkers. For example, different primary producers synthesize a wide range of phyto-sterols that are in turn consumed by herbivores [50]. However, most consumers either convert these phytosterols to cholesterol or simply oxidize the phytosterols for energy [50], so in this case, a great deal of source information can get reduced to only one molecule in the consumer. Similarly, different primary producer taxa can have very

different elemental ratios (e.g., C:N, C:P, N:P, etc.) but due to consumer homeostasis, the potential wide variation in resources gets collapsed down to much less variability in consumers [51]. Algal pigments have also been used as dietary tracers, but many algal pigments degrade very rapidly (< 1 hour) in consumer guts [52].

Conclusions

Using FA based analyses within the Bayesian framework [42,43] previously developed for SI data [10,11] may to a large extent alleviate the underdetermined constraint and FA based analyses did not have a bias towards the prior assumption. Moreover, the FA-based approach enables much higher resolution of distinct producer groups than what is possible with SI, even if source-specific SI fractionation is known. Our results strongly support Fry's [8,13] hypothesis that the underdetermined constraint will tend to bias the outputs of SI based Bayesian mixing model solutions towards the prior generalist assumption. Therefore, one must always carefully consider whether a generalist outcome is actually an indication of model failure, rather than a real result [8,13–14]. In the SI cases analyzed for this study, the two isotope-based solutions were almost exactly intermediate between the prior assumption and the correct value. Sensitivity analyses indicated this bias was equally due to uncertainty for the consumers and resources. Both of these sources of uncertainty caused flat posterior distributions that made it exceedingly difficult to discern among potential food sources. Uncertainty associated with the trophic fractionation assumption was also a very large source of output error. These results indicate the underdetermined constraint may be insurmountable when only a few SI are applied, and the potential food resources are poorly resolved.

The main sources of error for FA based analyses are likely to be model misspecification, such as not accounting for important resources in the consumer-resource library, and deviations between the FA profiles used in our consumer-resource library and the actual profiles of consumers utilizing similar resources in natural systems. Finally, a FA based approach can only be used in cases where it is possible to generate a suitable consumer-resource library file based on controlled feeding trials.

Supporting Information

S1 Fig. Stream t-DOC concentrations.
(DOC)

S1 File. FASTAR mixing model script and 4 dependent data files.
(ZIP)

S1 Table. Resource and consumer fatty acid composition.
(DOC)

S2 Table. Test of alternative priors.
(DOC)

Acknowledgments

We thank the Protistology & Aquatic Ecology culture collection at Ghent University, who supplied most of the diatom cultures used in this analysis. We also thank JS Yeung and ME Marian for their help in the lab, and two anonymous reviewers for their constructive criticisms of an earlier version of this study.

Author Contributions

Conceived and designed the experiments: AWEG MTB GWH EJW. Performed the experiments: MTB APB CWB MJK DCMN JP JLR US SJT GA. Analyzed the data: AWEG MTB. Contributed reagents/materials/analysis tools: AWEG GWH EJW. Wrote the paper: AWEG MTB GWH EJW APB CWB MJK DCMN JP JLR US SJT GA.

References

1. Brett MT, Kainz MJ, Taipale SJ, Seshan H (2009) Phytoplankton, not allochthonous carbon, sustains herbivorous zooplankton production. Proc Natl Acad Sci USA 106: 21197–21201. doi: 10.1073/pnas.0904129106 PMID: 19934044
2. Knisely K, Geller W (1986) Selective feeding of four zooplankton species on natural lake phytoplankton. Oecologia 69: 86–94.
3. DeMott WR (1988) Discrimination between algae and artificial particles by freshwater and marine copepods. Limnol Oceanogr 33: 397–408.
4. Brett MT, Wiackowski K, Lubnow FS, Müller-Solger A, Elser JJ, Goldman CR (1994) Species-dependent effects of zooplankton on planktonic ecosystem processes in Castle Lake, California. Ecology 75: 2243–2254.
5. Dalsgaard J, St John M, Kattner G, Müller-Navarra DC, Hagen W (2003) Fatty acid trophic markers in the pelagic marine environment. Adv Mar Biol 46: 225–340. PMID: 14601414
6. Peterson BJ, Fry B (1987) Stable isotopes in ecosystem studies. Ann Rev Ecol Syst 18: 293–320.
7. Parnell AC, Inger R, Bearhop S & Jackson AL (2010) Source partitioning using stable isotopes: coping with too much variation. PLoS ONE 5: e9672. doi: 10.1371/journal.pone.0009672 PMID: 20300637
8. Fry B (2013) Alternative approaches for solving underdetermined isotope mixing problems. Mar Ecol Prog Ser 472: 1–13.
9. Phillips DL, Gregg JW (2003) Source partitioning using stable isotopes: coping with too many sources. Oecologia 136: 261–269. PMID: 12759813
10. Moore JW, Semmens BX (2008) Incorporating uncertainty and prior information into stable isotope mixing models. Ecol Lett 11: 470–480. doi: 10.1111/j.1461-0248.2008.01163.x PMID: 18294213
11. Ward EJ, Semmens BX, Schindler DE (2010) Including source uncertainty and prior information in the analysis of stable isotope mixing models. Env Sci Tech 44: 4645–4650.
12. Semmens BX, Ward EJ, Parnell AC, Phillips DL, Bearhop S, Inger R, et al. (2013) Statistical basis and outputs of stable isotope mixing models: Comment on Fry (2013). Mar Ecol Prog Ser 490: 285–289.
13. Fry B (2013) Minmax solutions for underdetermined isotope mixing problems: Reply to Semmens et al. (2013). Mar Ecol Prog Ser 490: 291–294.
14. Brett MT (2014) Resource polygon geometry predicts Bayesian stable isotope mixing model bias. Mar Ecol Prog Ser 514: 1–12.
15. Brett MT, Müller-Navarra DC, Ballantyne AP, Ravet JL, Goldman CR (2006) *Daphnia* fatty acid composition reflects that of their diet. Limnol Oceanogr 51: 2428–2437.
16. Burns CW, Brett MT, Schallenberg M (2011) A comparison of the trophic transfer of fatty acids in freshwater plankton by cladocerans and calanoid copepods. Freshw Biol 56: 889–903.
17. Taipale SJ, Kainz MJ, Brett MT (2011) Diet-switching experiments show rapid accumulation and preferential retention of highly unsaturated fatty acids in *Daphnia*. Oikos 120: 1674–1682.
18. Brett MT, Müller-Navarra DC, Persson J (2009) Crustacean zooplankton fatty acid composition. Pages 115–146 in Chapter 6 in Arts MT, Brett MT, and Kainz M, editors. Lipids in Aquatic Ecosystems. Springer, New York.
19. Galloway AWE, Britton-Simmons KH, Duggins DO, Gabrielson PW, Brett MT (2012) Fatty acid signatures differentiate marine macrophytes at ordinal and family ranks. J Phycol 48: 956–965.
20. Taipale SJ, Strandberg U, Peltomaa E, Galloway AWE, Ojala A, Brett MT (2013) Fatty acid composition as biomarkers of freshwater microalgae: analysis of 37 strains of microalgae in 22 genera and in 7 classes. Aquat Microb Ecol 71: 165–178.
21. Brett MT, Müller-Navarra DC (1997) The role of essential fatty acids in aquatic food web processes. Freshw Biol 38: 483–499.
22. Ballantyne AP, Brahney J, Fernandez D, Lawrence CL, Saros J, Neff JC, et al. (2011) Biogeochemical response of alpine lakes to a recent increase in dust deposition in the Southwestern, US. Biogeosciences 8: 2689–2706.

23. Dethier MN, Sosik EA, Galloway AWE, Duggins DO, Simenstad CA (2013) Addressing assumptions: variation in stable isotopes and fatty acids in marine macrophytes can confound conclusions of food web studies. Mar Ecol Prog Ser 478: 1–14.
24. Nosrati K, Govers G, Semmens BX, Ward EJ (2014) A mixing model to incorporate uncertainty in sediment fingerprinting. Geoderma 217: 173–180.
25. Iverson SJ, Field C, Bowen WD, Blanchard W (2004) Quantitative fatty acid signature analysis: a new method of estimating predator diets. Ecol Monogr 74: 211–235.
26. Vuorio K, Meili M, Sarvala J (2006) Taxon-specific variation in the stable isotopic signatures ($\delta^{13}C$ and $\delta^{15}N$) of lake phytoplankton. Freshw Biol 51: 807–822.
27. Plummer M (2003) JAGS: A program for analysis of Bayesian graphical models using Gibbs sampling. In Proceedings of the 3rd International Workshop on Distributed Statistical Computing (DSC 2003). March (pp. 20–22).
28. R Core Team (2013). R: A language and environment for statistical computing. R Foundation for Statistical Computing, Vienna, Austria. ISBN 3-900051-07-0, URL http://www.R-project.org/
29. Semmens BX, Ward EJ, Moore JW, Darimont CT (2009) Quantifying inter-and intra-population niche variability using hierarchical Bayesian stable isotope mixing models. PloS ONE 4: e6187. doi: 10.1371/journal.pone.0006187 PMID: 19587790
30. Post DM (2002) Using stable isotopes to estimate trophic position: Models, methods, and assumptions. Ecology 83: 703–718.
31. Bond AL, Diamond AW (2011) Recent Bayesian stable-isotope mixing models are highly sensitive to variation in discrimination factors. Ecol Appl 21: 1017–1023. PMID: 21774408
32. Bunn SE, Leigh C, Jardine TD (2013) Diet-tissue fractionation of $\delta^{15}N$ by consumers from streams and rivers. Limnol Oceanogr 58: 765–773.
33. Parnell AC, Phillips DL, Bearhop S, Semmens BX, Ward EJ, Moore JW, et al. (2013) Bayesian stable isotope mixing models. Environmetrics 24: 387–399.
34. Graham CT, Harrison SSC, Harrod C (2014) Differences in the contributions of dietary water to the hydrogen stable isotope ratios of cultured Atlantic salmon and Arctic charr tissues. Hydrobiologia 721: 45–55.
35. Caut S, Angulo E, Courchamp F (2009) Variation in discrimination factors ($\Delta^{15}N$ and $\Delta^{13}C$): the effect of diet isotopic values and applications for diet reconstruction. J Appl Ecol 46: 443–453.
36. Strandberg U, Taipale SJ, Kainz MJ, Brett MT (2014) Retroconversion of docosapentaenoic acid (n-6): an alternative pathway for biosynthesis of arachidonic acid in *Daphnia magna*. Lipids 49: 591–595. doi: 10.1007/s11745-014-3902-y PMID: 24715497
37. Persson J, Vrede T (2006) Polyunsaturated fatty acids in zooplankton: variation due to taxonomy and trophic position. Freshw Biol 51: 887–900.
38. Adams TS, Sterner RW (2000) The effect of dietary nitrogen content on trophic level $\delta^{15}N$ enrichment. Limnol Oceanogr 45: 601–607.
39. Prado P, Carmichael RH, Watts SA, Cebrian J, Heck KL Jr (2012) Diet-dependent $\delta^{13}C$ and $\delta^{15}N$ fractionation among sea urchin *Lytechinus variegatus* tissues: implications for food web models. Mar Ecol Prog Ser 462: 175–190.
40. Cole JJ, Carpenter SR, Kitchell J, Pace ML, Solomon CT, Weidel B (2011) Strong evidence for terrestrial support of zooplankton in small lakes based on stable isotopes of carbon, nitrogen, and hydrogen. Proc Natl Acad Sci USA 108: 1975–1980. doi: 10.1073/pnas.1012807108 PMID: 21245299
41. Milke LM, Bricelj VM, Parrish CC (2006) Comparison of early life history stages of the bay scallop, *Argopecten irradians*: effects of microalgal diets on growth and biochemical composition. Aquaculture 260: 272–289.
42. Galloway AWE, Eisenlord ME, Dethier MN, Holtgrieve G, Brett MT (2014) Quantitative estimates of resource utilization by an herbivorous isopod using a Bayesian fatty acid mixing model. Mar Ecol Prog Ser 507: 219–232.
43. Galloway AWE, Taipale SJ, Hiltunen M, Peltomaa E, Strandberg U, Brett MT, et al. (2014) Diet specific biomarkers show that high quality phytoplankton fuel herbivorous zooplankton in large boreal lakes. Freshw Biol 59: 1902–1915.
44. McCarthy MD, Lehman J, Kudela R (2013) Compound-specific amino acid $\delta^{15}N$ patterns in marine algae: tracer potential for cyanobacterial vs. eukaryotic organic nitrogen sources in the ocean. Geochim Cosmochim Acta 103: 104–120.
45. Vadeboncoeur Y., Vander Zanden MJ, Lodge DM (2002) Putting the lake back together: Reintegrating benthic pathways into lake food web models. BioScience 52: 44–54.

46. Wassmann P, Duarte C, Agustí S, Sejr MK (2011) Footprints of climate change in the Arctic marine ecosystem. Glob Change Biol 17: 1235–1249.

47. Piepho M, Arts MT, Wacker A (2012) Species-specific variation in fatty acid concentrations of four phytoplankton species: does phosphorus supply influence the effect of light intensity or temperature? J Phycol 48: 64–73.

48. Schlechtriem C, Arts MT, Zellmer ID (2006) Effect of temperature on the fatty acid composition and temporal trajectories of fatty acids in fasting *Daphnia pulex* (Crustacea, Cladocera). Lipids 41: 397–400. PMID: 16808154

49. Müller-Navarra DC (2008) Food web paradigms: The biochemical view on trophic interactions. Int Rev Hydrobiol 93: 489–505.

50. Martin-Creuzburg D, Von Elert E (2004) Impact of 10 dietary sterols on growth and reproduction of *Daphnia galeata*. J Chem Ecol 30: 483–500. PMID: 15139303

51. Sterner RW, Elser JJ (2002) Ecological stoichiometry: the biology of elements from molecules to the biosphere. Princeton University Press.

52. Oechsler-Christensen B, Jónasdóttir SH, Henriksen P, Hansen PJ (2012) Use of phytoplankton pigments in estimating food selection of three marine copepods. J Plankt Res 34: 161–172.
